# The “Arab World” is Not a Useful Concept When Addressing Challenges to Public Health, Public Health Education, and Research in the Middle East

**DOI:** 10.3389/fpubh.2014.00030

**Published:** 2014-04-09

**Authors:** Iain Blair, Michal Grivna, Amer Ahmad Sharif

**Affiliations:** ^1^Institute of Public Health, College of Medicine and Health Sciences, United Arab Emirates University, Al Ain, United Arab Emirates; ^2^Education Division, Dubai Healthcare City, Dubai, United Arab Emirates

**Keywords:** public health, public health education, health research, Arab World

## Introduction

Interest in public health in the “Arab World” has intensified following the political and social changes that have affected the Middle East since 2010. A new text-book has been published (1), an international meeting has been held (2), a network of experts has been formed, and a special edition of major medical journal has been published (3). But how useful is the “Arab World” as a way of defining a geographical region in order to focus attention on the health challenges that it faces and in particular the challenges relating to public health research and education. In this brief essay, the authors argue that its usefulness is limited because the countries of the Arab World, however defined, are too heterogeneous to allow meaningful communal debate of their problems and solutions. As an alternative it is recommended that countries in the region form smaller more homogenous issue-specific groupings to discuss common challenges and action.

## Arab World

The preferred definition of the Arab World is the 22 member countries of the League of Arab States. The Eastern Mediterranean Region (EMR) of the World Health Organization (WHO) is a second important classification in which the Comoros, Mauritania, and Algeria are moved to the WHO Regional Office for Africa and three non-Arab countries are added: Afghanistan, Iran, and Pakistan. In addition, South Sudan is included. A third widely used classification is the World Bank’s Middle East and North Africa (MENA) grouping of 22 countries, which takes WHO EMR and excludes Sudan, Somalia, and Pakistan but adds Israel. Irrespective of which grouping is chosen, while there are “linguistic, political, historical, and socio-cultural links” between these nations there are also major dissimilarities at many levels and their heterogeneity is a major challenge (4). The region divides geographically into the Maghreb, the Mashreq, and the Gulf but the most important differences relate to levels of economic development, demography, population health status, inequity, political stability, history of conflict or war, presence of refugee or displaced populations, and health system organization. Given these differences does it make sense for countries to come together as the “Arab World” or WHO EMR to seek solutions to the specific challenges they face or would smaller issue-specific groups provide a more efficient forum for collaboration and joint action.

Attempts have already been made to define smaller more meaningful groups. On the basis of the World Bank income stratification, the Gulf States of Bahrain, Kuwait, Qatar, Saudi Arabia, United Arab Emirates, and Oman would be in a group of high income countries while Afghanistan, Comoros, Somalia, and South Sudan would be in a low income group while the remainder would be in a middle-income group. Furthermore, WHO already categorizes EMR countries into three groups based on health, health system performance, and expenditure on health (Table 1) and the Gulf States continue to work together through their health ministers’ executive board (5).

**Table 1:** **WHO EMR country classification**.

Group 1	Group 2	Group 3
Rapid social and economic development	Middle-income countries	Low population health outcomes
		Lack of resources for health
High income	Well-developed public health services	Political instability
	Limited resources	Other complex development challenges
Bahrain	Egypt	Afghanistan
Kuwait	Islamic Republic of Iran	Djibouti
Oman	Iraq	Pakistan
Qatar	Jordan	Somalia
Saudi Arabia	Lebanon	South Sudan
United Arab Emirates	Libya	Sudan
	Morocco	Yemen
	Occupied Palestinian Territory	
	Syrian Arab Republic	
	Tunisia	

*Source: World Health Organization Regional Office for the Eastern Mediterranean*.

## Challenges

The main challenges facing the countries of the region have been identified as inequities in health, rising exposure to health risks, increasing health care costs and unacceptably low levels of access to quality health care (6). To these may be added under-investment in research for health and the need to reform public health education (2) and the continuing challenges faced by countries with complex emergencies (7).

In terms of exposure to health risk factors and disparities in health status, the countries of the region vary (Table 2). Yemen and Sudan have not fully passed through the epidemiological transition and have a different set of key health risk factors and so it is unhelpful to group them with the other nations. This also applies to other EMR Group 3 countries (Afghanistan, Djibouti, Pakistan, and Somalia) and Comoros and Mauritania. These countries generally have not yet passed through the demographic transition either. The most recent data show that under-five mortality (per 1000) was 98.5 in Afghanistan, 80.9 in Djibouti, 85.9 in Pakistan, 147.4 in Somalia, 104 in South Sudan, and 73.1 in Sudan while the total fertility rate (per women) in Afghanistan, Pakistan, Somalia, South Sudan, and Sudan was 5.1, 3.5, 6.4, 6.7, and 3.9, respectively (8). However, with respect to risk factors and health status, amongst the other nations there may be enough similarities to justify a regional grouping.

**Table 2:** **Arab World/WHO EMR, life expectancy, GDP per capita, total expenditure on health, density of physicians, nurses and midwives, standardized mortality for selected conditions, main risk factors, selected countries, 2010**.

		United Arab Emirates	Lebanon	Morocco	Egypt	Oman	Yemen	Sudan
Life expectancy at birth, 2010, both sexes combined		76.3	77.5	72.7	70.6	75.9	65.8	68.8
GDP per capita		49005	9904	3082	2801	25536	1617	1234
Per capita total expenditure on health, US$		1640	622	186	137	598	88	104
Physicians per 10,000 population		14.7	36.5	6.1	7.7	19.5	3.0	3.7
Nurses and midwives per 10,000 population		26.0	29.1	9.0	13.8	19.5	7.2	10.0
Age-standardized death rate (per 100,000)	All causes	615	513	695	844	596	1068	799
	Communicable, maternal, neonatal, and nutritional disorders	45	37	118	80	83	469	373
	Non-communicable diseases	517	428	547	728	443	551	341
	Cardiovascular and circulatory diseases	295	248	239	441	241	331	132
	Neoplasms	73	97	106	71	56	62	70
	Injuries	52	47	30	35	70	47	85
	Diabetes	34	18	84	12	66	27	20
Main risk factors that account for the most disease burden (% DALYs)		High body-mass index, dietary risks, and high fasting plasma glucose	Dietary risks, smoking, high blood pressure, and high body- mass index	High body-mass index, dietary risks, and high fasting plasma glucose	Dietary risks, high blood pressure, and high body-mass index	High body-mass index, dietary risks, and high fasting plasma glucose	Suboptimal breastfeeding, childhood underweight, and dietary risks	Childhood underweight, household air pollution, and suboptimal breastfeeding

*Sources: Institute for Health Metrics and Evaluation (10), WHO Regional Office for the Eastern Mediterranean Region (8)*.

## Demography, Health Systems, and Complex Emergencies

However, the usefulness of a regional grouping is once more in doubt when demography and health systems are considered. The countries of EMR Group 1 which are also the countries of the Gulf Cooperation Council (GCC) have unique population structures with large expatriate populations relative to the size of their national populations. A typical population pyramid is that of the UAE where, 50% of the population are non-citizen males of working age (9). This population structure along with rapid population growth from high net inward migration has necessitated high and increasing government spending on healthcare. Again, in the UAE, which is typical of other GCC countries, total expenditure on health (THE) has averaged between 2 and 4% of gross domestic product (GDP) over the past 15 years. Since UAE GDP has more than trebled in this time from $100 billion to $380 billion this means that THE has also increased from $752 (per capita) in 2000 to $1640 in 2011. Of this, about 75% comes from government and the remaining 25% from private sources namely insurance and out-of-pocket payments. Government expenditure on health makes up about 9% of all government expenditure and has risen over fivefold from $1.7 billion in 2000 to $9.5 billion in 2011 easily outpacing both growth in population and GDP (11). In contrast, in EMR Group 2 countries, THE accounts for 6–8% of GDP, THE per capita is lower at $100–600 and out-of-pocket expenditure makes up a higher proportion of THE (56% in Lebanon, 58% in Egypt) (7). Jordan and Iraq are exceptions where although THE is low, only about a quarter is made up of out-of-pocket expenditure.

Health system strengthening is among the five strategic priorities identified by the WHO Regional Office for the Eastern Mediterranean for the next 5 years (along with progress on the Millenium Development Goals, action on non-communicable diseases, emergency preparedness, and emerging infectious diseases). Action to strengthen health systems inevitably translates to health system reform but because countries in the region face different issues, the solutions they seek are also different. In EMR Group 1 countries, health system reforms are designed to reduce government involvement in health and control expenditure by shifting payment and provision responsibilities to the private sector (12). In addition, in these countries there has been a rapidly growing private sector and this has demanded the development of new regulatory arrangements. On the other hand, EMR Group 2 countries have focused on universal coverage and reducing out-of-pocket health expenditure and risk of impoverishment from catastrophic medical expenses. The WHO EMR sub-divisions also have value when discussing the health consequences of war, conflict, and other complex emergencies, which tend to disproportionately affect Group 2 and Group 3 countries.

## National Health Research Systems

Population health improvement and reducing health disparities require good evidence, which in turn requires good research. Popular metrics for assessing health research include government expenditure on research and development as a percentage of GDP, number of scientific publications, number of patents, and number of researchers per million population. Even if useful, often these metrics are not available, but this has not prevented commentators from being highly critical of health research in the Middle East. Ten countries in the region (Bahrain, Jordan, Kuwait, Lebanon, Oman, Qatar, Saudi Arabia, Tunisia, United Arab Emirates, and Yemen) participated in a mapping exercise in 2006 (13) using available metrics, qualitative data from stakeholder interviews and document analysis. The results revealed deficiencies in National Health Research Systems (NHRS) in the areas of capacity, activity, governance, priorities, strategy, outcomes, and use of research and there followed a plea for all countries to strengthen their NHRS but in different ways. A more recent report has further criticized health research in the region’s countries although little new data was presented (14) while a further report from UAE presented a more optimistic view (15). But again it is not helpful to consider the region as homogenous whole with respect to health research. There is an obvious difference between EMR Group 1 countries which have the financial resources to carry out research but lack the higher education systems to generate new knowledge, EMR Group 2 countries which while not wealthy have mature education systems, good science and technology human resources, and good output of scientific publications and EMR Group 3 countries which have very limited financial and human resources for research (16). In addition, it is worth noting that EMR Group 1 countries score highly on metrics for innovation and use of information and communications technology. For example, the UAE is the highest-ranking Arab country in terms of its capacity for innovation and boasts the highest level of Internet availability. So once again attempting to diagnose problems and find solutions at the level of the Arab World is not helpful, a more sophisticated approach is required focusing on the needs of individual nations.

## Health Workforce and Public Health Education

The availability, accessibility, and quality of the health workforce has rightly been identified as the bedrock on which a strong health system should be built (17) but few countries in the region have good data on their health workforce, they differ widely in terms of a key metric (density of skilled health professionals) (8) and have very different recruitment and development needs. For example, the UAE relies heavily on overseas recruitment to fulfill its health workforce needs and expatriates comprise 87% of physicians, 88% of dentists, 94% of allied health professionals, and 99% of midwives and nurses and there is a need for effective planning and training and retention strategies to guarantee a sustainable health workforce in the future (18). Commentators have described the public health workforce in the Arab World as “weak” while admitting that data is lacking (19). Kronfol in a recent historical review of health professions’ education in the Arab world, while calling for improved training in public health and health services management, provides a more balanced view (20). He rightly shows that different countries face different problems and that solutions must be tailored to the needs of individual nations. This view is shared by Evashwick following a recent review of the extensive literature on the education of public health professionals. She comments that “relating public health education across national boundaries and education systems is complex given the diversity of public health education within a given country” (21). Countries in the region should conduct their own individual assessment of capacity within the public health workforce and design development programs to provide a sustainable and appropriate workforce for the future. Again as an example in the UAE, at the main public funded federal university, public health academics are already making changes to the nation’s public health education framework at both undergraduate and postgraduate levels (22, 23).

This is our final example of where attempting to analyze and solve problems at the level of the Arab World is unhelpful. Workforce needs, availability of programs, accreditation, quality, and expenditure are challenges that different countries will face at different times. Identifying, analyzing, and solving these problems must be viewed as work in progress for each individual nation and attempting a universal solution at the level of the Arab World is inappropriate.

## Conclusion

The Arab World is a disparate group of nations with very different health, public health, and health systems challenges. The idea that representatives of these nations can come together to debate and find common solutions has become fashionable recently but in reality this approach is unlikely to succeed. A more practical approach would be to analyze health problems and seek solutions at the level of smaller groups of similar nations that share common characteristics. The three WHO sub-divisions of EMR nations discussed in this paper or the GCC health ministers group are proposed as starting points.

Future meeting of these groups would have very different agendas. The high income countries of the Gulf would focus more on health systems reform, expenditure and quality, and less on universal coverage and out-of-pocket expenses. They would discuss the growing burden of obesity, diabetes, cardiovascular disease, and injury rather than polio elimination or under-five mortality. They would be more concerned with migrant and occupational health and less with the social determinants of health and the problems of conflict and displaced populations. Finally, they would be freed to consider health research needs, public health programs, health professions education, and workforce development.

At a time when there is an unprecedented vogue for attempting to find the solutions to health problems at a global or regional level it may be opportune to start to “think” as well as “act” a little closer to home.

## Author Contributions

All authors contributed to the ideas contained in this essay. Iain Blair produced the first draft. All authors refined the paper and edited the final version.

## Conflict of Interest Statement

The authors declare that the research was conducted in the absence of any commercial or financial relationships that could be construed as a potential conflict of interest.
